# Supplementary material for: Systemic and respiratory T-cells induced by seasonal H1N1 influenza protect against pandemic H2N2 in ferrets
Source: Commun Biol. 2020 Oct 9;3:564. doi: 10.1038/s42003-020-01278-5 (PMC7547016; doi:10.1038/s42003-020-01278-5)
Supplement: Supplementary file 1 — Supplementary Information [file 42003_2020_1278_MOESM1_ESM.pdf]

Supplementary Figures for

Systemic and respiratory T-cells induced by seasonal H1N1 influenza protect against pandemic H2N2 in ferrets

Koen van de Ven[1], Femke de Heij[1,3], Harry van Dijken[1], José A. Ferreira[2], Jørgen de Jonge[1#]

[1]Centre for Infectious Disease Control, National Institute for Public Health and the Environment (RIVM), Bilthoven, the Netherlands
[2]Department of Statistics, Informatics and Modelling, National Institute for Public Health and the Environment (RIVM), Bilthoven, The Netherlands.
[3]Present address: Princess Máxima Center for Pediatric Oncology, Utrecht, the Netherlands.

[#]Correspondence should be addressed to jorgen.de.jonge@rivm.nl

Supplementary Fig. 1

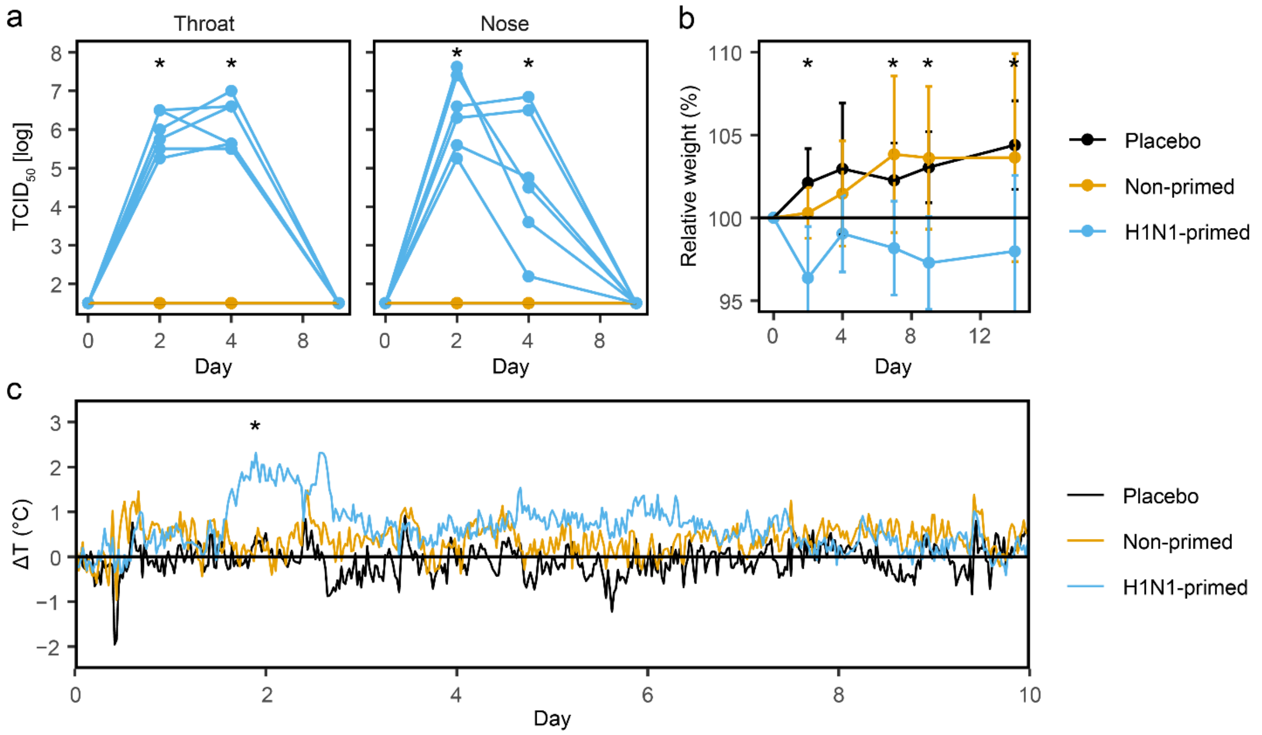

Supplementary Fig. 1: H1N1 viral replication and disease manifestation. **a)** Viral replication in throat and nose prior to infection (day 0) and 2, 4 and 7 days after infection with A/California/07/2009 (H1N1). Lines depict individual ferrets (n = 6). **b)** Relative changes in weight from the moment of H1N1 infection until day 14 (n = 6). Data are displayed as mean ± SD. **c)** Temperature displayed as mean deviation from baseline in 30 minute intervals from the day of H1N1 infection until day 10 (n = 5-6). Baseline temperature was calculated as the average temperature over 4 days prior to H1N1 infection. * indicates significant differences ($p < 0.05$) between non-primed and H1N1-primed groups after correction for multiple testing.

Supplementary Fig. 2

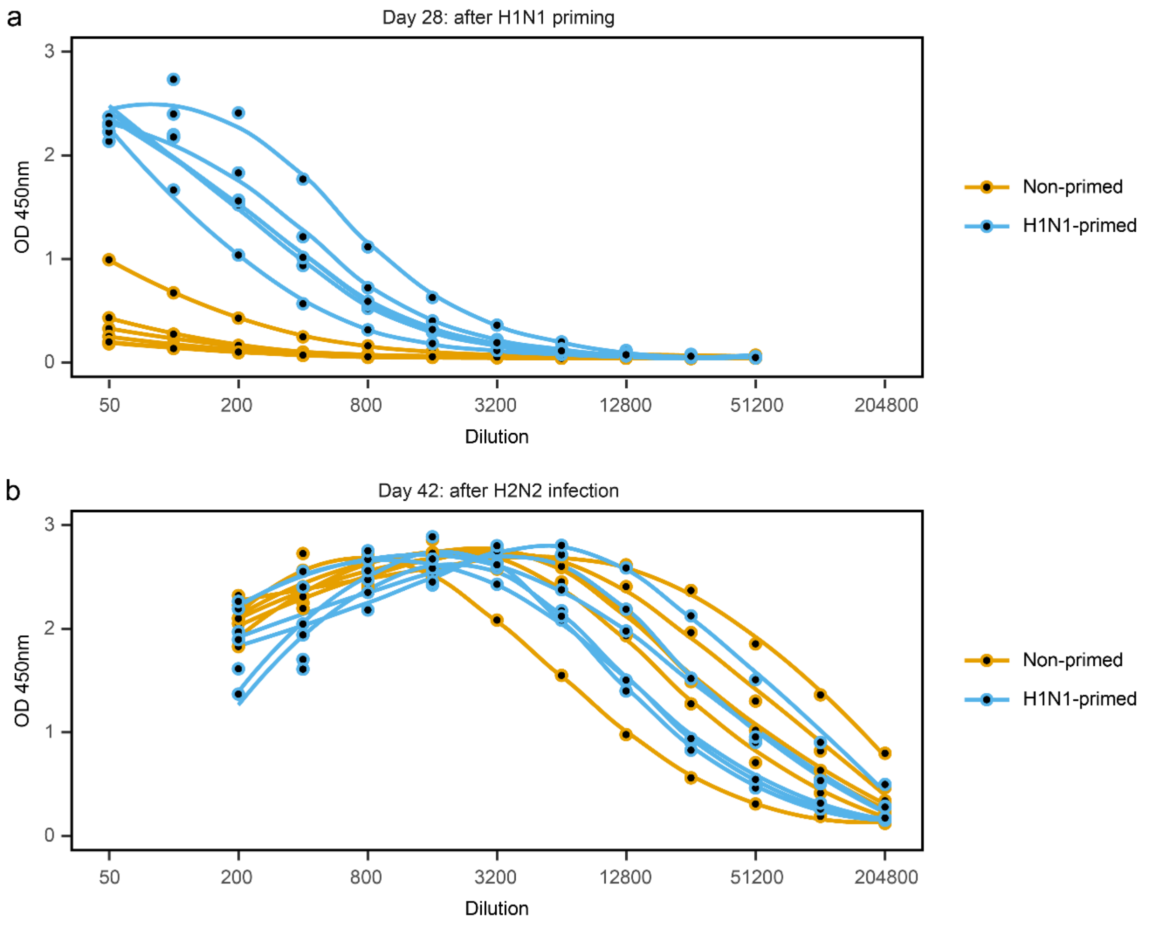

Supplementary Fig. 2: H1N1-priming induces low levels of H2-cross reactive antibodies. Sera from ferrets collected at **a)** day 28 (after H1N1 priming) and **b)** day 42 (14 days after H2N2 infection) were tested by ELISA for the presence of H2-binding IgG. Lines depict the smoothed curves of individual animals, which were calculated with Local Polynomial Regression Fitting. Points depict the actual measurements per individual animal.

Supplementary Fig. 3

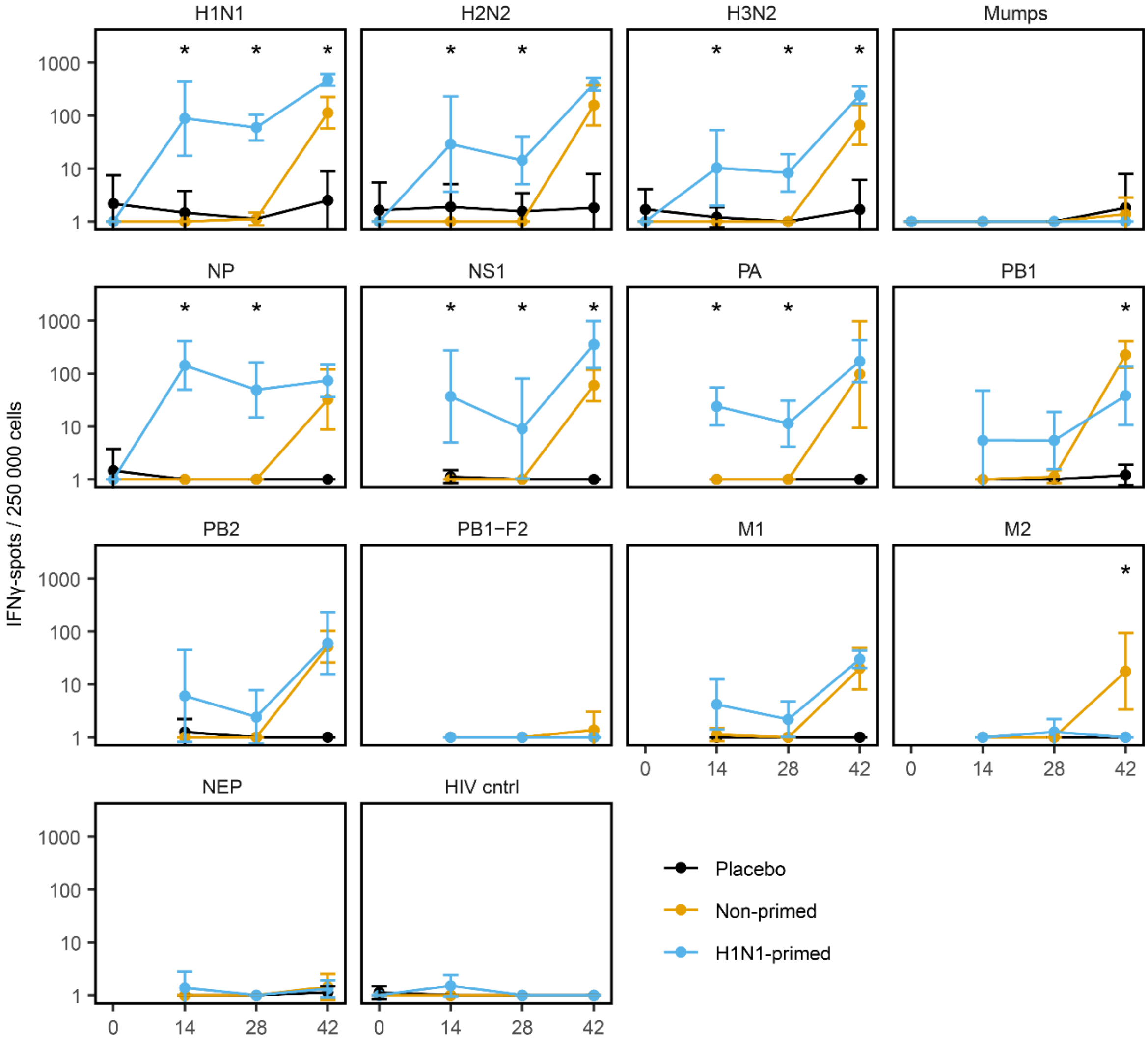

Supplementary Fig. 3: IFNγ responses to influenza peptide stimulations, measured in PBMCs by ELISpot over time. Blood samples were taken before (day 0) and two weeks after (day 14) H1N1 priming and before (day 28) and two weeks after (day 42) H2N2 infection. Lines represent means per groups of 5-6 animals ± SD. * indicates significant differences ($p < 0.05$) between non-primed and H1N1-primed groups after correction for multiple testing.

Supplementary Fig. 4

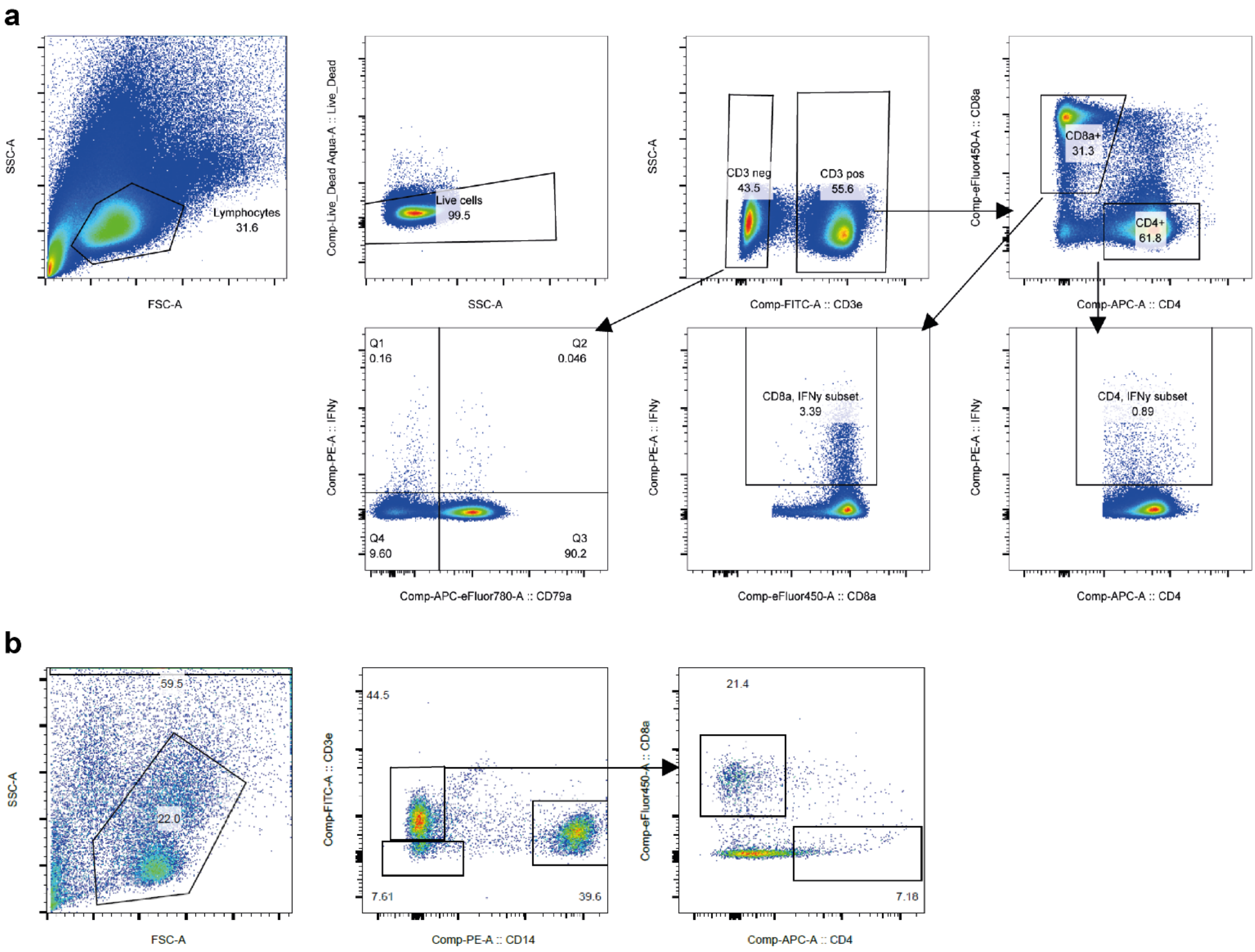

Supplementary Fig. 4: Gating strategy for flow cytometric analysis. **a)** Gating strategy for analysis of IFNγ responses. Plots show PBMCs stimulated with H2N2 influenza (A/Singapore/1/57) and are representative for other stimulations and tissues. **b)** Gating strategy of CD4 and CD8 T cells for Trucount analysis of broncho alveolar lavage (BAL) and nasal turbinates. The plots displayed are from the BAL of one animal, but are representative for other animals and tissues.
