# Supplementary material for: Systemic and respiratory T-cells induced by seasonal H1N1 influenza protect against pandemic H2N2 in ferrets
Source: Commun Biol. 2020 Oct 9;3:564. doi: 10.1038/s42003-020-01278-5 (PMC7547016; doi:10.1038/s42003-020-01278-5)
Supplement: Supplementary file 3 — Description of Additional Supplementary Files [file 42003_2020_1278_MOESM3_ESM.pdf]

Description of Additional Supplementary Files

File Name: Supplementary Data 1
Description: results from the statistical tests that support the main and supplemental figures.

File Name: Supplementary Data 2
Description: data supporting the main figures.
