# Supplementary material for: Systemic and respiratory T-cells induced by seasonal H1N1 influenza protect against pandemic H2N2 in ferrets
Source: Commun Biol. 2020 Oct 9;3:564. doi: 10.1038/s42003-020-01278-5 (PMC7547016; doi:10.1038/s42003-020-01278-5)
Supplement: Supplementary file 6 — Reporting Summary [file 42003_2020_1278_MOESM6_ESM.pdf]

nature research

Corresponding author(s): Jorgen de Jonge

Last updated by author(s): Aug 10, 2020

Reporting Summary

Nature Research wishes to improve the reproducibility of the work that we publish. This form provides structure for consistency and transparency in reporting. For further information on Nature Research policies, see Authors & Referees and the Editorial Policy Checklist .

Statistics

For all statistical analyses, confirm that the following items are present in the figure legend, table legend, main text, or Methods section.

n/a	Confirmed	
☐	☒	The exact sample size (*n*) for each experimental group/condition, given as a discrete number and unit of measurement
☒	☐	A statement on whether measurements were taken from distinct samples or whether the same sample was measured repeatedly
☐	☒	The statistical test(s) used AND whether they are one- or two-sided<br>*Only common tests should be described solely by name; describe more complex techniques in the Methods section.*
☒	☐	A description of all covariates tested
☐	☒	A description of any assumptions or corrections, such as tests of normality and adjustment for multiple comparisons
☐	☒	A full description of the statistical parameters including central tendency (e.g. means) or other basic estimates (e.g. regression coefficient) AND variation (e.g. standard deviation) or associated estimates of uncertainty (e.g. confidence intervals)
☐	☒	For null hypothesis testing, the test statistic (e.g. *F*, *t*, *r*) with confidence intervals, effect sizes, degrees of freedom and *P* value noted<br>*Give P values as exact values whenever suitable.*
☒	☐	For Bayesian analysis, information on the choice of priors and Markov chain Monte Carlo settings
☒	☐	For hierarchical and complex designs, identification of the appropriate level for tests and full reporting of outcomes
☐	☒	Estimates of effect sizes (e.g. Cohen's *d*, Pearson's *r*), indicating how they were calculated

Our web collection on statistics for biologists contains articles on many of the points above.

Software and code

Policy information about availability of computer code

Data collection	Mercury (version 4.91) software for collecting temperature data from loggers;<br>BD FACSDiva version 8.0.1
Data analysis	R version 3.6.0 with R-packages ggplot (v3.2.1) and coin (v1.2.1);<br>Flowjo (version 10) for analysis of flow data;<br>CTL immunospot (version 7.0.17.0) for analysis of Elispot plates;

For manuscripts utilizing custom algorithms or software that are central to the research but not yet described in published literature, software must be made available to editors/reviewers. We strongly encourage code deposition in a community repository (e.g. GitHub). See the Nature Research guidelines for submitting code & software for further information.

Data

Policy information about availability of data

All manuscripts must include a data availability statement. This statement should provide the following information, where applicable:
- Accession codes, unique identifiers, or web links for publicly available datasets
- A list of figures that have associated raw data
- A description of any restrictions on data availability

Results from the statistical analysis are available as Supplementary Data 1. All data supporting the main figures are available as Supplementary Data 2. This includes the GISAID identifiers for influenza sequences and all known influenza A epitopes from the IEDB at the day of retrieval. Note that data presented in Supplementary Data 2 has not been corrected for background responses (Elispot) or log-transformed (Elispot, HAI and VN titers). All raw data files are being stored in-house on backed-up servers and are available upon reasonable request to the corresponding author.

Field-specific reporting

Please select the one below that is the best fit for your research. If you are not sure, read the appropriate sections before making your selection.

- [x] Life sciences
- [ ] Behavioural & social sciences
- [ ] Ecological, evolutionary & environmental sciences

For a reference copy of the document with all sections, see nature.com/documents/nr-reporting-summary-flat.pdf

Life sciences study design

All studies must disclose on these points even when the disclosure is negative.

Sample size	In previous pilot experiments, the standard variation in the IFNg response of CD8 T-cells in PBMC's and spleen was approximately 2.5% in influenza infected animals after ex vivo virus stimulation. To be able to measure a difference between groups of 3.5% in the IFNg response in the CD8 T-cell subset, it was determined by sample size calculation that 6 animals per group were required (power=80; alpha=0.05).
Data exclusions	Temperature data from two animals was excluded due to faulty transponders. No other data were excluded.
Replication	The animal study was performed once. All immunological assays were performed once in singlets (Elispot, flow cytometry), duplo (hemagglutination assay), triplo (virus neutralization assay) or 6-plo (TCID50 for swabs and virus titer determination).
Randomization	Ferrets were semi-randomly allocated to groups by weight, to ensure average bodyweight was similar between groups
Blinding	Investigators were not blinded to the allocation of animals or analysis of the results as the investigators were supervising the correct administration of the virus suspension.

Reporting for specific materials, systems and methods

We require information from authors about some types of materials, experimental systems and methods used in many studies. Here, indicate whether each material, system or method listed is relevant to your study. If you are not sure if a list item applies to your research, read the appropriate section before selecting a response.

Materials & experimental systems

n/a	Involved in the study	
[ ]	[x]	Antibodies
[ ]	[x]	Eukaryotic cell lines
[x]	[ ]	Palaeontology
[ ]	[x]	Animals and other organisms
[x]	[ ]	Human research participants
[x]	[ ]	Clinical data

Methods

n/a	Involved in the study	
[x]	[ ]	ChIP-seq
[ ]	[x]	Flow cytometry
[x]	[ ]	MRI-based neuroimaging

Antibodies

Antibodies used	CD3ε-FITC (Biorad, clone CD3-12, cat. MCA1477F, lot 1608, 1:50 dilution);<br>CD4-APC (Sino biologicals, clone 02, cat. HG08FE1401, lot HS11DE0401, 1:50 dilution);<br>CD8-eFluor450 (eBioscience, clone OKT-08, cat. 48-0086-42, lot E10912-1633, 1:100 dilution);<br>CD14-PE (Invitrogen, clone Tük4, cat. MHCD1404, lot 1876081A, 1:100 dilution);<br>CD79a-APC/eFluor780 (eBioscience, clone HM47, cat. 12-0792-42, lot 4309673, 1:50 dilution);<br>IFNγ-PE (AbD Serotec (now Biorad), clone CC302, cat. MCA1783PE, lot 0912, 1:50 dilution);<br>Goat Anti-Ferret IgG (gamma)-HRP conjugate (Alpha Diagnostic, cat. 70530 1:5000 dilution)
Validation	CD4 is ferret specific and has been validated by Sino Biologicals. CD3ε and IFNγ are validated by Biorad. CD14 has been shown to be cross-reactive to mink (closely related to ferret) and has been validated in-house for ferrets. CD79a has been validated in-house. CD8 and IFNγ were originally shown to be cross-reactive to ferret by Rutigliano et al. (2008), Journal of Immunological Methods.

Eukaryotic cell lines

Policy information about cell lines

Cell line source(s)	Madin-Darby Canine Kidney (MDCKs) from ATCC
Authentication	Cell lines were not authenticated except for visual inspection of their morphology

Mycoplasma contamination	Standard practice is that cell-lines are tested for the absence of Mycoplasma prior to preparation of a cell bank. In addition, during use, cell lines are routinely tested.
Commonly misidentified lines (See ICLAC register)	N/A

Animals and other organisms

Policy information about studies involving animals; ARRIVE guidelines recommended for reporting animal research

Laboratory animals	Female ferrets (Mustela putorius furo), aged 12-18 months
Wild animals	Study did not involve wild animals
Field-collected samples	Study did not involve samples collected from the field
Ethics oversight	Committee on Animal Experimentation of the Antonie van Leeuwenhoek terrain (DEC-Alt, Bilthoven, the Netherlands)

Note that full information on the approval of the study protocol must also be provided in the manuscript.

Flow Cytometry

Plots

Confirm that:

- [x] The axis labels state the marker and fluorochrome used (e.g. CD4-FITC).
- [x] The axis scales are clearly visible. Include numbers along axes only for bottom left plot of group (a 'group' is an analysis of identical markers).
- [x] All plots are contour plots with outliers or pseudocolor plots.
- [x] A numerical value for number of cells or percentage (with statistics) is provided.

Methodology

Sample preparation

Lymphocyte isolation
Blood was collected in sodium-heparin coated Vacutainers (BD) and diluted 1:1 with PBS (Gibco) for density centrifugation on a 1:1 mixture of LymphoPrep (1.077 g/ml, Stemcell) and Lympholyte-M (1.0875 g/ml, Cedarlane). Cells were spun down for 30 minutes at 800x g (RT) and the interphase was washed twice using washing medium (RPMI1640 + 1%FBS). The cells were collected in stimulation medium (RPMI1640 + 10% FBS + 1x penicillin-streptomycin-glutamine [Gibco]) and counted using a hemocytometer.

Spleens were homogenized in a sieve using the plunger of a 10mL syringe after which the suspension was collected in a 50 ml tube. The tube was gently inverted to mix the suspension after which the tube was left for 1-2 minutes to let the larger debris sink. The upper 40ml of cell suspension was transferred to another tube and centrifuged for 5 min at 500x g. The pellet was resuspended in EDTA-supplemented washing medium (RPMI1640 + 1% FBS + 2mM EDTA (Invitrogen)) and put over a 100µm cell strainer. The resulting suspension was layered on top of Lympholyte-M and density centrifugation was performed in a similar manner as described for blood. All washing steps were performed with EDTA-supplemented medium to prevent agglutination of cells.

BAL was washed twice with washing medium and resuspended in stimulation medium for further use. Lungs were processed into small cubes of approximately 5mm3 and digested in 12ml of collagenase I (2.4mg/ml, Merck) and DNase I (1mg/ml, Novus Biologicals) for 30 minutes at 37oC while rotating. Following this, samples were further homogenized in a sieve using a 10 mL plunger and subsequently washed with EDTA-supplemented washing medium. The suspension was then filtered over a 70µm cell strainer and used for density centrifugation similar to the spleen.

Nasal turbinates (NT) were gently mashed in a sieve and subsequently filtered over a 70µm cell strainer. The resulting suspension was rested for 1 minute to allow the cartilage fragments to sediment. The suspension – excluding the sedimented cartilage fragments – was transferred to another tube and washed twice with EDTA-supplemented washing medium. The cells were then resuspended in 40% Percoll (GE Healthcare) and layered on top of 70% Percoll. Samples were centrifuged for 20 minutes at 500x g after which the interphase was collected and washed twice with EDTA-supplemented washing medium. Lymphocytes were resuspended in stimulation medium for further use.

IFNγ responses by flow cytometry
1-3 million lymphocytes were stimulated with virus at MOI 1 for 24 hours or H2N2 peptide pools for 8 hours. Golgiplug (BD) was added 6 hours before the end of the stimulation followed by storage o/n at 4°C. Cells were washed twice with FACS buffer (2mM EDTA, 0.5% BSA in PBS) and extracellular staining was performed in 100µl FACS buffer with live-dead aqua (Invitrogen), α-CD4-APC (02, Sino Biological) and α-CD8a-eFluor450 (OKT8, eBioscience) for 30 minutes at 4oC. After washing, cells were fixated and permeabilised with Foxp3/Transcription factor staining buffer set (eBioscience) according to the manufacturers protocol. Cells were then stained intracellularly with α-CD3e-FITC (CD3-12, Biorad), α-CD79a-APC/eFluor780 (eBioscience) and α-IFNγ-PE (CC302, Bioconnect) for 30 minutes at 4oC. After washing twice, the pellet was resuspended in FACS buffer and measured on a LSR Fortessa X-20 (BD). Data was analyzed using FlowJoTM Software V10 (BD).

Cell counts by flow cytometry
To reduce cell loss inherent to washing and centrifugation steps during staining, NT and BAL samples were stained using the non-centrifugation PerFix-NC kit (Beckman Coulter) according to the manufacturers protocol. In brief, cells were stained with α-CD4-APC, α-CD8a-eFluor450, and α-CD14-PE (Tük4; Thermo Fisher) for 30 minutes at RT. Subsequently, cells were fixated with 25µl Fixative Reagent for 15 minutes followed by permeabilization by the addition of 300µl of Permeabilizing Reagent containing α-CD3e-FITC and α-CD79a-APC/eFluor780. Cells were intracellularly stained for 30 minutes at RT, after which 3ml of Final Reagent was added to each tube. To concentrate the cells the tube was spun down (500x g, 5 min) and 2.5ml of the liquid was discarded while the pellet was resuspended in the remaining volume. 50µl of Coulter Flow-Count Fluorospheres (Beckman Coulter) was added to each sample and the sample was vortexed just before measurement on a LSR Fortessa X-20.

Instrument

LSRFortessa X-20

Software

Flowjo (BD) version 10; BD FACSDiva version 8.0.1

Cell population abundance

No samples were sorted

Gating strategy

For IFNy-population:
Lymphocytes (FSC vs SSC) > singlets (FSC-A vs FSC-H) > live cells (Live/dead aqua; invitrogen) > T cells (CD3-FITC vs SSC) > CD4 and CD8 T cells (CD4-APC vs CD8-eFluor450) > IFNy+ (CD8-eFluor450 vs IFNy-PE). CD79a-APC/eFluor780 was gated within the CD3-negative fraction, but was not used for further analysis.

For Trucount:
Lymphocytes (FSC vs SSC) > T cells (CD3-FITC vs CD14-PE) > CD4 and CD8 T cells (CD4-APC vs CD8-eFluor450). CD79a-APC/eFluor780 was gated within the CD3-negative fraction, but was not used for further analysis.

☒ Tick this box to confirm that a figure exemplifying the gating strategy is provided in the Supplementary Information.
